# The Association Between Endometriosis Treatments and Depression and/or Anxiety in a Population-Based Pathologically Confirmed Cohort of People with Endometriosis

**DOI:** 10.1089/whr.2023.0068

**Published:** 2023-11-20

**Authors:** Emma Goodwin, Helena Abreu do Valle, Amanda Nitschke, Joseph Puyat, Paul J. Yong, Gillian E. Hanley

**Affiliations:** ^1^ Department of Gynaecology and Obstetrics, Division of Gynaecologic Oncology, University of British Columbia, Vancouver, Canada.; ^2^ School of Population and Public Health, University of British Columbia, Vancouver, Canada.

**Keywords:** endometriosis, depression, anxiety, pelvic pain, surgery, reoperation

## Abstract

**Objective:**

Endometriosis patients have a high rate of co-occurring anxiety and depression. There is currently no literature investigating how this may affect endometriosis treatment and outcomes. This study examines the prevalence of depression and anxiety in a pathologically confirmed population-based endometriosis cohort and examines how endometriosis treatments and outcomes differ by the presence of co-occurring depression and/or anxiety.

**Methods:**

This retrospective cohort study using population-based administrative data sets included pathologically confirmed endometriosis patients identified from the complete pathology records of Vancouver Coastal Health Authority (British Columbia, Canada) between 2000 and 2008. These data were linked with population-based health data for follow-up to 2017. Bivariate analyses assessed differences between patients with depression and/or anxiety and those without. Odds ratios (ORs) were calculated to assess the odds of binary postsurgical outcomes.

**Results:**

Our final cohort consisted of 3815 patients. There were 603 patients (15.8%) with depression and/or anxiety. They were more likely to visit a physician for pelvic pain, more likely to take some hormonal medications, and more likely to fill prescription-level analgesics, including opioids both before and after surgery. They also had a significantly higher risk of reoperation for their endometriosis than people without co-occurring depression and/or anxiety (OR 1.32, 95% confidence interval [CI]: 1.07–1.61).

**Conclusion:**

Endometriosis patients with co-occurring depression and/or anxiety used more health services for pain, including prescription-level analgesics, and were more likely to have an endometriosis reoperation. We recommend that future study should aim to better understand the direction of this association between depression and/or anxiety and increased health services use.

## Introduction

Endometriosis affects 10% of biologically female people of reproductive age.^1^ It is a chronic estrogen-dependent inflammatory condition characterized by lesions resembling uterine endometrium (epithelium and stroma) found outside of the uterus, including elsewhere in the pelvis but can be disseminated in other parts of the body.^2^ Common symptoms of endometriosis include chronic pelvic pain, dysmenorrhea, dyspareunia, dyschezia/dysuria, pain after the menstrual period, and infertility,^3^ and thus endometriosis can have important consequences on a person's quality of life.^4^

Previous research has illustrated that endometriosis patients have a prevalence rate of ∼15% for depression and ∼30% for anxiety.^5–7^ However, results vary considerably, as some studies have reported prevalence rates of 55.7% for depression and 25.3% for anxiety in their samples.^8^ Hazard ratios are estimated to be ∼1.4 for any depressive disorder (95% confidence interval [CI]: 1.21–1.69) and 1.39 for anxiety disorders (95% CI: 1.14–1.71) when compared with age and sex-matched controls.^9^

Moreover, mice have been shown to exhibit higher levels of anxiety and depression, lower locomotion, and persistent pain hypersensitivity after induced endometriosis, suggesting that the endometriosis may somehow be predisposing to anxiety and depression.^10^ Other chronic and inflammatory conditions, such as rheumatoid arthritis and inflammatory bowel disease, have been studied in relation to mental health. Research showed that rheumatoid arthritis patients have a threefold increase in the prevalence of major depressive disorder compared with the general population.^11^ A population-based study in the United Kingdom reported that 17.5% of their inflammatory bowel disease cohort had a depressive disorder compared with 12.9% of their matched controls.^12^

Common treatments for endometriosis include surgery (excision or ablation of lesions, with or without hysterectomy/oophorectomy) and/or hormonal therapy. Endometriosis is an estrogen-dependent disease, and progestin-based medications are used to suppress estrogen including *via* suppression of the hypothalamic–pituitary–ovarian axis. Other medication treatments including gonadotropin-releasing hormone (GnRH) agonists, which target the GnRH receptor, also suppress the hypothalamic–pituitary–ovarian axis.^1^ However, recurrence of disease and incomplete alleviation of symptoms are relatively common with these treatments. Reoperation is not uncommon after surgical treatment, but specific rates vary considerably, with estimates ranging from ∼1% to 58%.^13,14^

Many previous studies examining the association between endometriosis and anxiety and depression have used self-reported endometriosis, or other non-gold standard diagnostic approaches. They have also not examined whether and how treatment may differ among endometriosis patients with comorbid depression and/or anxiety compared with those without comorbid depression and/or anxiety. Herein, we examine the prevalence of comorbid depression and/or anxiety in a cohort of surgically confirmed endometriosis patients.

We then examine prescription medication use (including hormonal treatments, prescription-level analgesics, and psychotropic medication use), physician visits for pelvic pain and endometriosis, and rates of reoperation after surgery and compare treatment received by those with comorbid depression and/or anxiety with those without.

In the general Canadian population, the estimated lifetime prevalence of depression is 11.2% and estimated lifetime prevalence of anxiety is 8.7%, with females at twice the risk of both conditions compared with males.^15,16^ We hypothesize that prevalence of comorbid depression and/or anxiety will be higher in this cohort, and that those with depression and/or anxiety will have higher health services use for endometriosis.

## Methods

We conducted a population-based retrospective cohort study of all people with endometriosis in the final diagnosis in their pathology report in the pathology database of Vancouver Coastal Health (population ∼1 million). We used the search terms “endometriosis,” “endometrioma,” and/or “endometriotic cyst,” and examined all pathology reports with those search terms between the years 2000 and 2008. These data were then linked to population-based administrative data sets containing information on hospitalizations, and physician visits between 1998 and 2017.^17–23^

These data were linked to the BC PharmaNet,^24^ which contains records of every prescription dispensed in an outpatient setting to identify all relevant medications used by the patients in our cohort between 1998 and 2017. Ethical approval was obtained from the University of British Columbia Clinical Research Ethics Board. Access to data provided by the Data Steward(s) is subject to approval, but can be requested for research projects through the Data Steward(s) or their designated service providers. All inferences, opinions, and conclusions are those of the authors, and do not reflect the opinions or policies of the Data Stewards.

### Cohort

Inclusion criteria for our cohort include undergoing surgery in Vancouver Coastal Health authority during our study period with a subsequent pathologically confirmed diagnosis of endometriosis listed on their pathology report. The surgery that resulted in inclusion in our cohort is referred to as their index surgery, which represents the first surgery during our study period that resulted in a diagnosis of pathologically confirmed endometriosis. Each individual in the cohort is unique. Patients were excluded if (1) their age was >80 or <15 years; (2) they were diagnosed with a gynecological malignancy; or (3) they had any history of schizophrenia, manic disorder, bipolar disorder, or psychosis, given that our primary interest was in anxiety and depression before the index surgery.

### Defining depression and anxiety

To determine which patients had comorbid depression and/or anxiety, we examined the physician visits and hospital data for all individuals in our cohort. We used a lookback period of 2 years before the index surgery to find any evidence of depression and/or anxiety in this cohort. A person was defined as having comorbid depression and/or anxiety if they had two diagnostic codes for depression and/or anxiety in 1 year, at least 30 days apart in the 2 years before their index surgery. Supplementary Table S1 details the diagnostic codes used to identify comorbid depression and/or anxiety.

Using these data sets codes to identify depression and/or anxiety has previously been validated and found to have high rates of accuracy in identifying psychiatric conditions.^25,26^ We present the data with depression and/or anxiety as a composite outcome because of the presence of a diagnostic code (ICD-9 code 50B) that is specific to British Columbia that groups the two conditions together. However, we stratified the cohort according to diagnostic codes for each condition individually and looked for significant differences by depression or anxiety, as well as a code that indicates either depression or anxiety (50B code). The results were similar across groups, so for parsimony we present the results by the composite outcome. The stratified results are presented in Supplementary Tables S2–S4.

### Endometriosis treatments

We examined reoperations for endometriosis occurring >90 days after the index surgery. These surgeries were identified by the Canadian Classification of Health Interventions (CCI) procedure codes. This system separately identifies each procedure performed in the same surgery, and thus there are often multiple relevant procedure codes in the same surgery. We defined a reoperation as any surgery with one or more of the endometriosis-related procedure codes outlined in Supplementary Table S5.

Also examined were visits to a physician with diagnostic codes for pelvic pain (ICD-10-CM R10.2 or R10.3, ICD-9-625) or endometriosis (ICD-10-CM- N80.X, ICD-9-617), hormonal medication use, prescription-level analgesic use, which included nonsteroidal anti-inflammatory drugs (NSAIDs) and opioids, and psychotropic medication use (Supplementary Table S6). We used a 3-month washout period postsurgery to exclude routine postsurgical physician visits, prescriptions, and surgical complications.

Hormonal medication use was divided into groups based on the hormones in the medication prescribed. Combined estrogen and progestogen (as a combined medication prescription or two prescriptions, one for an estrogen and one for a progestogen) was separated into hormonal contraceptives, used to suppress endometriosis, and hormone replacement therapy (HRT). Estrogen and progestogen combination in this category that included a levonorgestrel intrauterine system (*n* = 69) was classified as hormonal contraceptives. For other HRTs, we also present data on use of systemic unopposed estrogen, local estrogen, and progestogen only. Finally, GnRH agonists are presented as medications to treat endometriosis.

### Covariates

From the pathology reports, we collected data on the following: (1) the clinical history listed on the pathology report, which included whether the patient was presenting with pain, a suspected mass/cancer, adenomyosis, a suspected endometrioma/endometriotic cyst, infertility, suspected other endometriosis, or suspected fibroids at their index surgery; (2) co-occurring conditions listed on the pathology report including endometrial hyperplasia, adenomyosis, and fibroids; (3) location of the endometriosis (*e.g.*, endometrioma, superficial ovarian endometriosis, endometriosis elsewhere in the pelvis, or endometriosis outside the pelvis); and (4) age at the time of index surgery.

Age was also used as an approximation for menopause status. Although there is variation around menopausal age, we used age <50 years as a proxy for premenopausal status and age ≥50 years as postmenopausal status because the average age of natural menopause is ∼51 years.^27^

Through linkage of the pathology reports with the population-based administrative data, we obtained data on (1) neighborhood household income, as defined by the income band the patient was in during the year of their pathology report; (2) information on all procedures that were performed during the index surgery (identified using CCI codes above), including surgical approach (laparoscopic, open, and vaginal); and (3) surgical history of the patients to determine which patients had a previous unilateral or bilateral oophorectomy or a previous endometriosis surgery, again defined through CCI codes, >45 days before their index surgery.

### Statistical analysis

Bivariate analyses were performed to assess potential differences between those with depression and/or anxiety and those without depression and/or anxiety. Descriptive comparisons were completed using *t*-tests for continuous numerical variables and chi square tests for categorical variables. Wilcoxon rank sum test was used to compare medians of numeric variables that were not normally distributed. Odds ratios (ORs) to assess the odds of binary postsurgical treatments were calculated using logistic regression models. The covariates defined above were considered for the adjusted models, except in cases wherein there were too few patients with positive data (*e.g.*, co-occurring adenomyosis, endometrial hyperplasia, and unilateral oophorectomy).

To prevent skewed results, patients who underwent a reoperation for endometriosis were removed from the cohort when the ORs were calculated for all other outcomes. Given that we included individuals who had an incidental diagnosis of endometriosis and that the relationship between depression and anxiety and subsequent endometriosis treatments may differ in this group, we also conducted a sensitivity analysis on the individuals who did not have any history of endometriosis in the form of physician visits, previous surgery, or indication for index surgery. All statistical tests were conducted using R Studio (version 4.0.5) and a *p*-value <0.05 was considered significant.

## Results

**FIG. 1.**
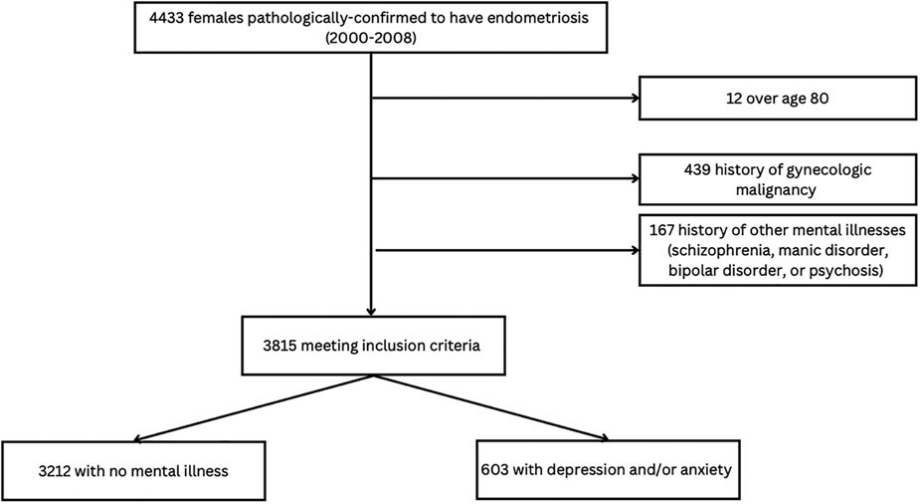
Inclusion and exclusion criteria flow chart.

Our cohort consisted of 4433 unique patients who underwent surgery with a pathology report that confirmed a pathological diagnosis of endometriosis between January 1, 2000, and December 31, 2008. After applying exclusion criteria, there were 3815 patients. Of these, 3212 (84.2%) patients had no depression and/or anxiety and 603 (15.8%) met our definition of comorbid depression and/or anxiety in the 2 years before their index surgery (Fig. 1). From the stratified results, we determined that the prevalence of depression was 5.6%, anxiety was 4.1%, and anxiety/depression (code 50B) was 6.0% (Supplementary Tables S2–S4).

As given in Table 1, there were no significant differences in the age, income quintile, year of pathology report, menopausal status (menopause was defined by age >50 years), procedures performed during the index surgery, or surgical approach between the two groups. The only exception was that patients with depression and/or anxiety were less likely to have “other” procedures during their index surgery (*p* < 0.001). These include inspection of the uterus and surrounding structures, ovarian cystectomy, partial excision of the fallopian tube, trachelectomy, and uterine nerve ablation. Patients with depression and/or anxiety were more likely to have a clinical history of pain as the indication for their surgery (*p* < 0.001).

**Table 1.** Baseline Patient Characteristics at Time of Index Surgery

	No mental illness (*N* = 3212)	*Depression and/or anxiety (* *N* * = 603)*	*p*
Age at time of pathology report, mean (SD)	39.1 (9.31)	38.8 (8.92)	0.528
SES quintile, *n* (%)			
1	653 (20.3)	108 (17.9)	0.36
2	668 (20.8)	124 (20.6)	
3	597 (18.6)	130 (21.6)	
4	643 (20.0)	126 (20.9)	
5	648 (20.2)	115 (19.1)	
Missing	≤5	0 (0)	
Year of pathology report, median [min, max]	2010 [2000, 2010]	2010 [2000, 2010]	
Premenopause (age <50 years)	2854 (88.9)	540 (89.6)	0.666
Postmenopause (age ≥50 years)	358 (11.1)	63 (10.4)	0.666
Procedures performed during index surgery, *n* (%)			
Index surgery for endometriosis	1591 (49.5)	298 (49.4)	0.995
Hysterectomy	407 (12.7)	100 (16.6)	0.019
Bilateral oophorectomy	880 (27.4)	171 (28.4)	0.664
Salpingectomy	190 (5.9)	31 (5.1)	0.419
Biopsy	223 (6.9)	45 (7.5)	0.833
Adhesiolysis	561 (17.5)	111 (18.4)	0.827
Excision	520 (16.2)	106 (17.6)	0.382
Ablation	277 (8.6)	61 (10.1)	0.357
Other	614 (19.1)	92 (15.3)	0.018
Surgical approach, *n* (%)			
Laparoscopic	717 (22.3)	140 (23.2)	0.667
Abdominal	573 (17.8)	107 (17.7)	1
Vaginal	269 (8.4)	57 (9.5)	0.43
Laparoscopic/vaginal combination	142 (4.4)	34 (5.6)	0.229
Missing	592 (18.4)	104 (17.2)	0.527
Indication for surgery, *n* (%)			
Mass/suspected cancer	542 (16.9)	79 (13.1)	0.025
Pain	478 (14.9)	136 (22.6)	<0.001
Endometrioma	364 (11.3)	41 (6.8)	0.001
Other endometriosis	952 (29.6)	190 (31.5)	0.383
Infertility	249 (7.8)	29 (4.8)	0.014
Cyst	532 (16.6)	77 (12.8%)	0.023
Adenomyosis	17 (0.5)	≤5	0.384
Fibroids	380 (11.8)	61 (10.1)	0.255
Surgical history in the 2 years before the index surgery, *n* (%)			
Prior unilateral oophorectomy	248 (7.7)	44 (7.3)	0.677
Prior bilateral oophorectomy	43 (1.3)	10 (1.7)	0.67
Prior endometriosis surgery >45 days before path report	345 (10.7)	104 (17.2)	<0.001
Diagnoses from pathology report at index surgery, *n* (%)			
Endometriosis	3212 (100)	603 (100)	NA
Endometrioma/endometriotic cyst	1202 (37.4)	164 (27.2)	<0.001
Superficial endometriosis on the ovary	532 (16.6)	81 (13.4)	0.063
Endometriosis in the pelvis	2199 (68.5)	472 (78.3)	<0.001
Endometriosis outside the pelvis	123 (3.8)	23 (3.8)	1
Endometrial hyperplasia	47 (1.5)	9 (1.5)	1
Adenomyosis	369 (11.5)	66 (10.9)	0.753
Fibroids	693 (21.6)	128 (21.2)	0.891

SD, standard deviation; SES, socioeconomic status.

In addition, these patients were less likely to have a mass/suspected cancer, infertility, cysts, and endometrioma as an indication for surgery (*p* < 0.05). A significantly higher proportion of those with depression and/or anxiety had an endometriosis surgery >45 days before the index surgery (*p* < 0.001). During the index surgery, patients with depression and/or anxiety were less likely to have endometriomas/endometriotic cysts diagnosed in their pathology report, but more likely to have other endometriosis in the pelvis (*p* < 0.001).

Before the index surgery, patients with depression and/or anxiety were more likely to be seeing a physician for pelvic pain and endometriosis, including for two visits in 1 year, at least 30 days apart (Table 2, *p* < 0.001). They were more likely to be using combined estrogens and progestogens, both hormonal contraceptives and HRT (*p* < 0.001), and GnRH agonists (*p* < 0.05). Furthermore, these patients were significantly more likely to be filling prescriptions for NSAIDs (54.5% vs. 67.8%, *p* < 0.001) and opioids (61.1% vs. 75.3%, *p* < 0.001). Significance remained when looking at the number of prescriptions and the number of prescription days dispensed for both NSAIDs and opioids.

**Table 2.** Endometriosis Treatments Received in the 2 Years Before the Index Surgery, Stratified by the Presence or Absence of Comorbid Depression and/or Anxiety in the 2 Years Before the Index Surgery

	No mental illness (*N* = 3212)	*Depression and/or anxiety (* *N* * = 603)*	*p*
Physician visits, *n* (%)			
At least one visit for pelvic pain	1504 (46.8)	378 (62.7)	<0.001
At least two visits for pelvic pain (30 days apart)	746 (23.2)	227 (37.6)	<0.001
At least one visit for endometriosis	977 (30.4)	238 (39.5)	<0.001
At least two visits in 1 year for endometriosis (30 days apart)	453 (14.1)	117 (19.4)	0.001
Hormonal medications (any prescription), *n* (%)			
Estrogens and progestogens (any combination)	1143 (35.6)	293 (48.6)	<0.001
Hormonal contraceptives	1024 (31.9)	253 (42.0)	<0.001
HRT	119 (3.7)	40 (6.6)	0.001
Systemic estrogens only	67 (2.1)	15 (2.5)	0.638
Progestogens	277 (8.6)	54 (9.0)	0.852
Local estrogens	65 (2.0)	16 (2.7)	0.406
GnRH agonists	267 (8.3)	79 (13.1)	<0.001
Prescription-level analgesics			
NSAIDs			
Any prescription, *n* (%)	1750 (54.5)	409 (67.8)	<0.001
No. of prescriptions, median [min, max]	2.00 [1.00, 73.0]	3.00 [1.00, 47.0]	<0.001
No. of days dispensed, median [min, max]	24.0 [2.00, 2320]	40.0 [3.00, 1430]	<0.001
Opioids			
Any prescription, *n* (%)	1961 (61.1)	454 (75.3)	<0.001
No. of prescriptions, median [min, max]	2.00 [1.00, 363]	3.00 [1.00, 356]	<0.001
No. of days dispensed, median [min, max]	8.00 [1.00, 3610]	15.0 [1.00, 2510]	<0.001
Psychotropics (any prescription), *n* (%)			
Anticonvulsants	69 (2.1)	39 (6.5)	<0.001
Antidepressants (other than SRIs, 2 years before)	183 (5.7)	118 (19.6)	<0.001
Antidepressants (other than SRIs, 5 years before)	282 (8.8)	164 (27.2)	<0.001
SRIs (2 years before)	157 (4.9)	294 (48.8)	<0.001
SRIs (5 years before)	297 (9.2)	334 (55.4)	<0.001
Benzodiazepines	448 (13.9)	255 (42.3)	<0.001

GnRH, gonadotropin-releasing hormone; HRT, hormone replacement therapy; NSAID, nonsteroidal anti-inflammatory drug; SRI, serotonin reuptake inhibitor.

After the index surgery, 872 (22.9%) patients in this cohort had an endometriosis-related reoperation (Table 3). Those with depression and/or anxiety were more likely to have an endometriosis-related reoperation (26.5% vs. 22.2%, *p* < 0.05). A significantly higher proportion of those with depression and/or anxiety were visiting a physician for pelvic pain both in the 3- to 24-month period (30.7% vs. 20.2%, *p* < 0.001) and 2- to 5-year period (29.2% vs. 20.0%, *p* < 0.001) after the index surgery.

**Table 3.** Endometriosis Treatments Received After the Index Surgery, Stratified by the Presence or Absence of Comorbid Depression and/or Anxiety in the 2 Years Before the Index Surgery

	No mental illness (*N* = 3212)	*Depression and/or anxiety (* *N* * = 603)*	*p*
Reoperation for endometriosis, *n* (%)	712 (22.2)	160 (26.5)	0.024
Missing, *n* (%)	61 (1.9)	10 (1.7)	
Physician visits: 3–24 months postindex surgery			
Pelvic pain, *n* (%)	648 (20.2)	185 (30.7)	<0.001
No. of visits for pain, median [min, max]	1.00 [1.00, 20.0]	2.00 [1.00, 30.0]	0.005
Endometriosis, *n* (%)	625 (19.5)	129 (21.4)	0.299
No. of visits for endometriosis, median [min, max]	2.00 [1.00, 20.0]	2.00 [1.00, 25.0]	0.421
Physician visits: 2–5 years postindex surgery			
Pelvic pain, *n* (%)	643 (20.0)	176 (29.2)	<0.001
No. of visits for pain, median [min, max]	2.00 [1.00, 45.0]	2.00 [1.00, 77.0]	0.060
Endometriosis, *n* (%)	554 (17.2)	118 (19.6)	0.189
No. of visits for endometriosis, median [min, max]	2.00 [1.00, 29.0]	2.00 [1.00, 25.0]	0.300
Hormonal medications (any prescription postindex surgery), *n* (%)			
Estrogens and progestogens (any combination)	1211 (37.7)	248 (41.1)	0.123
Hormonal contraceptives	765 (23.8)	135 (22.4)	0.48
HRT	446 (13.9)	113 (18.7)	0.002
Systemic estrogen	322 (10.0)	69 (11.4)	0.327
Progestogens	314 (9.8)	80 (13.3)	0.012
Local estrogens	451 (14.0)	126 (20.9)	<0.001
GnRH agonists	324 (10.1)	58 (9.6)	0.781
By previous use, *n* (%)			
Estrogens and progestogens (any combination)	521 (16.2)	118 (19.6)	0.05
Hormonal contraceptives	406 (12)	90 (14.9)	0.143
HRT	36 (1.1)	23 (3.8)	<0.001
Systemic estrogens	41 (1.3)	≤5	0.471
Progestogens	39 (1.2)	7 (1.2)	1
Local estrogens	24 (0.7)	7 (1.2)	0.429
GnRH agonists	53 (1.7)	13 (2.2)	
Prescription-level analgesic use, 3–24 months postindex surgery			
NSAIDs			
Any prescription, *n* (%)	689 (21.5)	215 (35.7)	<0.001
No. of prescriptions, median [min, max]	1.00 [1.00, 16.0]	1.00 [1.00, 16.0]	0.054
No. of prescription days dispensed, median [min, max]	20.0 [1.00, 1060]	30.0 [2.00, 1020]	0.004
Opioids			
Any prescription, *n* (%)	702 (21.9)	233 (38.6)	<0.001
No. of prescriptions, median [min, max]	2.00 [1.00, 776]	3.00 [1.00, 2750]	<0.001
No. of prescription days dispensed, median [min, max]	8.00 [1.00, 590]	20.0 [1.00, 575]	<0.001
Prescription-level analgesic use, 2–5 years postindex surgery			
NSAIDs			
Any prescription, *n* (%)	1062 (33.1)	259 (43.0)	<0.001
No. of prescriptions, median [min, max]	1.00 [1.00, 33.0]	2.00 [1.00, 66.0]	<0.001
No. of prescription days dispensed, median [min, max]	20.0 [1.00, 1060]	30.0 [2.00, 1020]	0.001
Opioids			
Any prescription, *n* (%)	1033 (32.2)	307 (50.9)	<0.001
No. of prescriptions, median [min, max]	2.00 [1.00, 776]	3.00 [1.00, 2750]	<0.001
No. of prescription days dispensed, median [min, max]	8.00 [1.00, 1140]	20.0 [1.00, 1110]	<0.001
Psychotropics (any prescription), *n* (%)			
Anticonvulsants	478 (14.9)	174 (28.9)	<0.001
Antidepressants (other than SRIs)	669 (20.8)	267 (44.3)	<0.001
SRIs	801 (24.9)	378 (62.7)	<0.001
Benzodiazepines	1007 (31.4)	354 (58.7)	<0.001

However, there was no significant difference between the two groups in visits to a physician for endometriosis. In terms of hormonal medications, those with depression and/or anxiety were more likely to use estrogens–progestogens for HRT, progestogens, and local estrogens, but not hormonal contraceptive estrogens–progestogens, systemic estrogens, or GnRH agonists.

In the 3–24 months postsurgery, 35.7% of patients who experienced depression and/or anxiety were filling prescriptions for NSAIDs compared with 21.5% of those with no mental illness (*p* < 0.001). Those with depression and/or anxiety also had a significantly higher number of NSAID prescription days dispensed (20.0 days vs. 30.0 days, *p* < 0.05). For opioids, patients with depression and/or anxiety were more likely to have a prescription (21.9% vs. 38.6%, *p* < 0.001), they also had a higher number of prescriptions in the time period (2.00 [1–776] vs. 3.00 [1–2750], *p* < 0.001), and more prescription days dispensed (8.00 [1–590] vs. 20.00 [1–575], *p* < 0.001).

In the 2–5 years postsurgery, these outcomes remained significant, with a higher proportion of those with depression and/or anxiety taking NSAIDs or opioids, having a higher number of prescriptions, and more prescription days dispensed. Not surprisingly, rates of use of psychotropic medications were higher among those with co-occurring depression and/or anxiety.

Several postsurgery binary outcomes were further explored using ORs. The unadjusted logistic regression models showed that the odds of reoperation, visiting a physician for pelvic pain, being prescribed combined estrogens–progestogens for HRT, local estrogens, NSAIDs, and opioids were all statistically significantly higher among those with depression and/or anxiety. The odds of a prescription for systemic estrogens, combined estrogens and progestogens for hormonal contraceptives, progestogens only, and GnRH agonists were not significantly different based on the presence of co-occurring depression and/or anxiety.

**FIG. 2.**
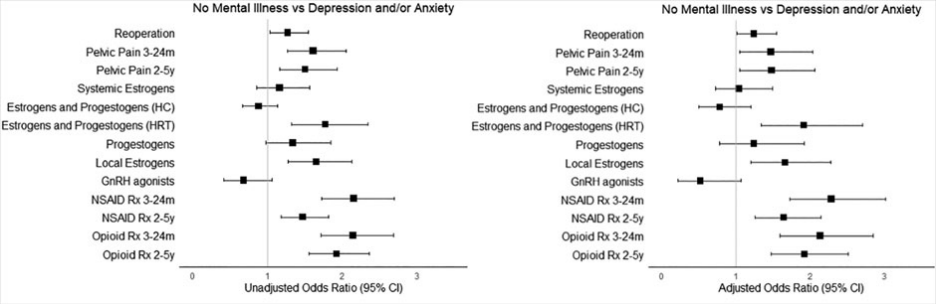
Unadjusted and adjusted ORs for postsurgical binary outcomes of interest. The adjusted models account for age, income, clinical indication for pain, mass/cancer, cyst, endometrioma/endometriotic cyst, infertility, other endometriosis, and fibroids, comorbid conditions at index surgery (adenomyosis, and fibroids), index surgery type, and surgical approach. HC, hormonal contraceptives; GnRH, gonadotropin-releasing hormone; HRT, hormone replacement therapy; NSAID, nonsteroidal anti-inflammatory drug; ORs, odds ratios; Rx, prescription.

As shown in Figure 2, after adjustment, odds of local estrogen use remained significantly higher in those with depression and/or anxiety (1.63, 95% CI: 1.17–2.24), as well as odds of estrogens and progestogens for HRT (2.03, 95% CI: 1.41–2.88). In the 3- to 24-month period after the index surgery, physician visits for pain (1.49, 95% CI: 1.06–2.08), filling a NSAID (22.32, 95% CI: 1.74–3.08), and filling an opioid prescription (2.06, 95% CI: 1.53–2.77) were significantly higher among those with co-occurring depression and/or anxiety.

In the 2- to 5-year period, odds of a physician visits for pain (1.45, 95% CI: 1.01–2.04), filling an NSAID (1.60, 95% CI: 1.22–2.10), and filling an opioid prescription (1.79, 95% CI: 1.36–2.34) were all significantly higher among those who had depression and/or anxiety. The odds of reoperation were also significant (1.32, 95% CI: 1.07–1.61) for those with depression and/or anxiety in the adjusted model.

In the sensitivity analysis of those diagnosed with endometriosis incidentally, the OR results were fairly consistent in direction and effect size with those in the main analysis but did not reach statistical significant due to the small sample size (*n* = 417; 358 with no mental illness and 59 with depression and/or anxiety) (Supplementary Fig. S1 and Supplementary Table S7).

## Discussion

In this population-based cohort of pathologically confirmed endometriosis patients, we reported a prevalence rate of 15.8% comorbid depression and/or anxiety in the 2-year before index surgery. These 15.8% of patients were more likely to have pain listed as an indication for their surgery and were more likely to have seen physicians for pelvic pain and endometriosis before their index surgery; to have had a prior endometriosis surgery; and to have filled medications for some combined estrogens and progestogens, GnRH agonists, NSAIDs, and opioids before their index surgery.

They were more likely to use many of these treatments after index surgery as well. Specifically, estrogens–progestogens for HRT, progestogens and local estrogens were significantly more likely to be used by the group with depression and/or anxiety. There was no longer a statistically significant difference in visits to a physician for endometriosis, use of combined estrogens and progestogens as hormonal contraceptives, and use of GnRH agonists.

In addition, the patients with depression and/or anxiety were more likely to be visiting a physician for pelvic pain and using both NSAIDs and opioids up to 5 years after their index surgery. This was true even after adjustment for covariates. Notably, there was also an increased risk for endometriosis-related reoperation in those with comorbid depression and/or anxiety.

Previous studies have reported varied prevalence rates of mental illness among endometriosis patients, including prevalence rates both lower and higher than what we report. For example, Gao et al. used a similar population-based approach to show that 3.1% of their cohort of patients who were diagnosed with endometriosis had an anxiety or stress-related disorder. In addition, 3.1% had a depressive disorder.^6^ Another study found that 29% of their sample had anxiety symptoms, 14.5% had depressive symptoms, 12.9% had both.^7^ Our prevalence rate is similar to that of Cavaggioni et al., who reported an anxiety prevalence of 10.8% and depression prevalence of 5.4%.^28^

Our results are also consistent with other research suggesting that pelvic pain is associated with anxiety and/or depression. We reported that people with depression and/or anxiety were seeing physicians more often for pelvic pain, and taking more analgesic medications, both before and after they had surgery than those without depression and/or anxiety. Similarly, Warzecha et al. reported that endometriosis patients with pelvic pain had a significantly higher rate of depressive symptoms than endometriosis patients without pelvic pain.^5^

Another study found that those with endometriosis-associated pelvic pain were experiencing lower quality of life and higher anxiety and depression than both asymptomatic endometriosis patients and healthy controls.^29^ Patients with depression and/or anxiety had a higher odds of using hormonal medications for HRT. Previous literature has found a higher risk for depression and anxiety in those who undergo surgical menopause, especially in younger populations; however, we reported no difference in bilateral oophorectomy rates, both in the index surgery and before the index surgery by depression and/or anxiety.^30,31^

This study is strengthened by its use of a large pathologically confirmed population-based cohort of endometriosis patients, along with 5 complete years of follow-up data to assess health care use and endometriosis treatments. It is also unique in addressing the association between anxiety and/or depression and treatment for endometriosis received. Although this focus on treatment is unique, given the observational nature of the data, we cannot comment on causality.

It seems likely that the people with endometriosis and comorbid depression and/or anxiety were suffering from greater severity of endometriosis symptoms, given their increased use of services before the index surgery, particularly with respect to pelvic pain. It is also possible that the patients with comorbid depression and/or anxiety are more likely to suffer from central sensitization, which can cause/worsen chronic pelvic pain, as an association between depression, anxiety, and central sensitization has previously been reported.^32^

Further limitations of this research include those common to working with population-based administrative data. We are limited by the data that are collected, and in BC this excludes race and/or ethnicity, gender, access to psychological services not covered by Medical Services Plan, body mass index, and education level. Our decision to require two diagnostic codes in a 12-month period (separated by 30 days) to classify a patient as having depression and/or anxiety was done to reduce false positives in that group; however, this is a trade-off with increasing the rate of false negatives, and the high use of psychotropic medications in the group without depression and/or anxiety suggests some misclassification in the exposure groups.

This misclassification would serve to bias toward finding fewer differences in treatment use between those with and without depression and/or anxiety and thus is a conservative bias. Also, we cannot determine from BC PharmaNet data whether or not the prescribed medications were taken by the patient, only whether or not they filled a prescription. Finally, we are also unable to generalize these results to patients with endometriosis who have not had surgery, as they are likely different from those included in this cohort that required an index surgery for cohort entry given the pathological confirmation of endometriosis.

We recommend that future studies address the relationship between depression and anxiety and pain in endometriosis patients, including trying to elucidate the biological or psychosocial mechanisms underpinning this association. Our results emphasize the need to treat endometriosis as a systemic condition. Given the high prevalence of depression and/or anxiety and the association between depression and/or anxiety and physician visits for pain, hormonal medication use, and prescription-level analgesic use suggesting that these patients may be experiencing worse symptoms, clinicians should consider incorporating depression and anxiety into their biopsychosocial assessment of endometriosis and chronic pelvic pain patients.

## Supplementary Material

Supplemental data

Supplemental data

Supplemental data

Supplemental data

Supplemental data

Supplemental data

## Abbreviations Used

CCI: Canadian Classification of Health Interventions
CI: confidence interval
GnRH: gonadotropin-releasing hormone
HRT: hormone replacement therapy
NSAID: nonsteroidal anti-inflammatory drug
OR: odds ratio
SD: standard deviation
SES: socioeconomic status
SRI: serotonin reuptake inhibitor
