# Supplementary material for: The Association Between Endometriosis Treatments and Depression and/or Anxiety in a Population-Based Pathologically Confirmed Cohort of People with Endometriosis
Source: Womens Health Rep (New Rochelle). 2023 Nov 20;4(1):551–61. doi: 10.1089/whr.2023.0068 (PMC10664573; doi:10.1089/whr.2023.0068)
Supplement: Supplemental data [file Suppl_TableS1.docx]

**Supplementary Table 1:** Diagnostic codes for mental health disorders

| Diagnosis group | ICD-10-CA | ICD-9 |
| --- | --- | --- |
| **Mental Health Condition** | | |
| Depression | F32-39 | 296.2-296.36; 311; 50B* |
| Anxiety disorder | F40-43 | 300; 50B* |
| **Mental Health Disorders Controlled For** | | |
| Alcohol/drug disorder | F10-F19 | 291-293 303 |
| Schizophrenia | F20-29 | 295 |
| Bipolar disorder | F30-31 | 296.0-296.16; 296.4-296.9 |
| Personality disorder | F60-69 | 301 |

*code is specific to British Columbia
