# Supplementary material for: The Association Between Endometriosis Treatments and Depression and/or Anxiety in a Population-Based Pathologically Confirmed Cohort of People with Endometriosis
Source: Womens Health Rep (New Rochelle). 2023 Nov 20;4(1):551–61. doi: 10.1089/whr.2023.0068 (PMC10664573; doi:10.1089/whr.2023.0068)
Supplement: Supplemental data [file Suppl_TableS2-S4.zip › SupplementaryTable2.docx]

**Supplementary Table 2.** Baseline patient characteristics at time of index surgery. (SES – Socioeconomic Status)

|  | **No Mental Illness (reference group) (N=3212)** | **Depression Only (N=217)** | **P-value** | **Anxiety Only (N=156)** | **P-value** | **Code 50B Only (N=230)** | **P-value** | |
| --- | --- | --- | --- | --- | --- | --- | --- | --- |
| Age at time of pathology report, mean (SD) | 39.1 (9.31) | 38.0 (9.52) | 0.115 | 38.2 (8.74) | 0.247 | 40.0 (8.36) | 0.118 | |
| SES Quintile |  |  |  |  |  |  |  | |
| 1 | 653 (20.3%) | 43 (19.8%) | 0.967 | 30 (19.2%) | 0.201 | 35 (15.2%) | 0.422 | |
| 2 | 668 (20.8%) | 46 (21.2%) |  | 30 (19.2%) |  | 48 (20.9%) |  | |
| 3 | 597 (18.6%) | 43 (19.8%) |  | 41 (26.3%) |  | 46 (20.0%) |  | |
| 4 | 643 (20.0%) | 45 (20.7%) |  | 29 (18.6%) |  | 52 (22.6%) |  | |
| 5 | 648 (20.2%) | 40 (18.4%) |  | 26 (16.7%) |  | 49 (21.3%) |  | |
| Missing | <=5 | 0 (0%) |  | 0 (0%) |  | 0 (0%) |  | |
| Year of pathology report |  |  |  |  |  |  | |  |
| Median [Min, Max] | 2010 [2000, 2010] | 2010 [2000, 2010] |  | 2010 [2000, 2010] |  | 2000 [2000, 2010] |  | |
|  |  |  |  |  |  |  |  | |
| Premenopause (Age < 50) | 2854 (88.9%) | 195 (89.9%) | 0.729 | 141 (90.4%) | 0.643 | 204 (88.7%) | 1 | |
| Postmenopause (Age >= 50) | 358 (11.1%) | 22 (10.1%) | 0.729 | 15 (9.6%) | 0.643 | 26 (11.3%) | 1 | |
| **Procedures performed during the index surgery** |  |  |  |  |  |  |  | |
| Index surgery for endometriosis | 1591 (49.5%) | 112 (51.6%) | 0.601 | 72 (46.2%) | 0.458 | 114 (49.6%) | 1 | |
| Hysterectomy | 407 (12.7%) | 40 (18.4%) | 0.0351 | 24 (15.4%) | 0.413 | 36 (15.7%) | 0.258 | |
| Bilateral Oophorectomy | 880 (27.4%) | 54 (24.9%) | 0.47 | 35 (22.4%) | 0.21 | 82 (35.7%) | 0.01 | |
| Salpingectomy | 190 (5.9%) | 13 (6.0%) | 1 | 9 (5.8%) | 1 | 9 (3.9%) | 0.237 | |
| Biopsy | 223 (6.9%) | 17 (7.8%) | 0.862 | 16 (10.3%) | 0.16 | 12 (5.2%) | 0.343 | |
| Adhesiolysis | 561 (17.5%) | 36 (16.6%) | 0.664 | 30 (19.2%) | 0.682 | 45 (19.6%) | 0.638 | |
| Excision | 520 (16.2%) | 45 (20.7%) | 0.067 | 33 (21.2%) | 0.123 | 28 (12.2%) | 0.121 | |
| Ablation | 277 (8.6%) | 18 (8.3%) | 0.853 | 21 (13.5%) | 0.0649 | 22 (9.6%) | 0.791 | |
| Other | 614 (19.1%) | 28 (12.9%) | 0.0167 | 33 (21.2%) | 0.549 | 31 (13.5%) | 0.0283 | |
| **Surgical Approach** |  |  |  |  |  |  |  | |
| Laparoscopic | 717 (22.3%) | 63 (29.0%) | 0.0279 | 35 (22.4%) | 1 | 42 (18.3%) | 0.176 | |
| Abdominal | 573 (17.8%) | 39 (18.0%) | 1 | 29 (18.6%) | 0.895 | 39 (17.0%) | 0.803 | |
| Vaginal | 269 (8.4%) | 20 (9.2%) | 0.76 | 16 (10.3%) | 0.498 | 21 (9.1%) | 0.783 | |
| Laparoscopic/vaginal combination | 142 (4.4%) | 8 (3.7%) | 0.734 | 16 (10.3%) | 0.00151 | 10 (4.3%) | 1 | |
| Missing | 592 (18.4%) | 32 (14.7%) | 0.204 | 17 (10.9%) | 0.0225 | 55 (23.9%) | 0.049 | |
| **Indication for Surgery** |  |  |  |  |  |  |  | |
| Mass/Cancer | 542 (16.9%) | 21 (9.7%) | 0.00747 | 20 (12.8%) | 0.224 | 38 (16.5%) | 0.963 | |
| Pain | 478 (14.9%) | 54 (24.9%) | <0.001 | 33 (21.2%) | 0.0436 | 49 (21.3%) | 0.0118 | |
| Endometrioma | 364 (11.3%) | 10 (4.6%) | 0.00305 | 15 (9.6%) | 0.594 | 16 (7.0%) | 0.0528 | |
| Other Endometriosis | 952 (29.6%) | 66 (30.4%) | 0.869 | 57 (36.5%) | 0.0805 | 67 (29.1%) | 0.93 | |
| Infertility | 249 (7.8%) | 9 (4.1%) | 0.0695 | 10 (6.4%) | 0.645 | 10 (4.3%) | 0.0782 | |
| Cyst | 532 (16.6%) | 25 (11.5%) | 0.0638 | 23 (14.7%) | 0.626 | 29 (12.6%) | 0.14 | |
| Adenomyosis | 17 (0.5%) | 0 (0%) | 0.565 | 0 (0%) | 0.74 | <=5 | 1 | |
| Fibroids | 380 (11.8%) | 19 (8.8%) | 0.208 | 11 (7.1%) | 0.0907 | 31 (13.5%) | 0.523 | |
| **Surgical History** |  |  |  |  |  |  |  | |
| Unilateral oophorectomy | 248 (7.7%) | 14 (6.5%) | 0.5 | 14 (9.0%) | 0.696 | 16 (7.0%) | 0.702 | |
| Bilateral oophorectomy | 43 (1.3%) | $\leq5$ | 0.83 | $\leq5$ | 0.79 | $\leq5$ | 0.45 | |
| Endometriosis surgery >45 days before pathology report | 345 (10.7%) | 48 (22.1%) | <0.001 | 21 (13.5%) | 0.35 | 35 (15.2%) | 0.0473 | |
| **Pathology Report** |  |  |  |  |  |  |  | |
| Endometriosis | 3212 (100%) | 217 (100%) | NA | 156 (100%) | NA | 230 (100%) | NA | |
| Endometrioma/endometriotic cyst | 1202 (37.4%) | 48 (22.1%) | <0.001 | 55 (35.3%) | 0.644 | 61 (26.5%) | 0.00118 | |
| Endometriosis in ovary - non-endometrioma/ endometriotic cyst | 532 (16.6%) | 29 (13.4%) | 0.255 | 17 (10.9%) | 0.0784 | 35 (15.2%) | 0.66 | |
| Other endometriosis in the pelvis | 2199 (68.5%) | 178 (82.0%) | <0.001 | 115 (73.7%) | 0.196 | 179 (77.8%) | 0.0038 | |
| Other endometriosis outside the pelvis | 123 (3.8%) | 9 (4.1%) | 0.957 | <=5 | 0.854 | 9 (3.9%) | 1 | |
| Endometrial Hyperplasia | 47 (1.5%) | <=5 | 0.722 | <=5 | 0.617 | 6 (2.6%) | 0.278 | |
| Adenomyosis | 369 (11.5%) | 22 (10.1%) | 0.62 | 15 (9.6%) | 0.555 | 29 (12.6%) | 0.684 | |
| Fibroids | 693 (21.6%) | 39 (18.0%) | 0.243 | 23 (14.7%) | 0.0528 | 66 (28.7%) | 0.0149 | |
