# Supplementary material for: The Association Between Endometriosis Treatments and Depression and/or Anxiety in a Population-Based Pathologically Confirmed Cohort of People with Endometriosis
Source: Womens Health Rep (New Rochelle). 2023 Nov 20;4(1):551–61. doi: 10.1089/whr.2023.0068 (PMC10664573; doi:10.1089/whr.2023.0068)
Supplement: Supplemental data [file Suppl_TableS2-S4.zip › SupplementaryTable3.docx]

**Supplementary Table 3**. Endometriosis treatments received in the two years before the index surgery, stratified by the presence or absence of comorbid depression and/or anxiety in the two years prior to the index surgery. (GnRH – Gonadotropin Releasing Hormone, NSAID – Non-steroidal Anti-inflammatory Drug, SRI – Serotonin Reuptake Inhibitor)

|  | **No Mental Illness (reference group) (N=3212)** | **Depression only (N=217)** | **P-value** | **Anxiety only (N=156)** | **P-value** | **Code 50B only (N=230)** | **P-value** |  |
| --- | --- | --- | --- | --- | --- | --- | --- | --- |
|  |  |  |  |  |  |  |  | |
| **Physician visits** |  |  |  |  |  |  |  | |
| At least one visit for pelvic pain | 1504 (46.8%) | 148 (68.2%) | <0.001 | 104 (66.7%) | <0.001 | 126 (54.8%) | 0.0234 |  |
| At least two visits for pelvic pain (30 days apart) | 746 (23.2%) | 95 (43.8%) | <0.001 | 62 (39.7%) | <0.001 | 70 (30.4%) | 0.0163 |  |
| At least one visit for endometriosis | 977 (30.4%) | 93 (42.9%) | <0.001 | 68 (43.6%) | <0.001 | 77 (33.5%) | 0.369 |  |
| At least two visits for endometriosis (30 days apart) | 453 (14.1%) | 54 (24.9%) | <0.001 | 27 (17.3%) | 0.317 | 36 (15.7%) | 0.581 |  |
| **Hormonal medications (any prescription)** |  |  |  |  |  |  |  |  |
| Systemic Estrogens | 67 (2.1%) | <=5 | 1 | 7 (4.5%) | 0.086 | <=5 | 0.907 |  |
| Estrogens and progestogens | 1143 (35.6%) | 111 (51.2 %) | <0.001 | 81 (51.9%) | <0.001 | 101 (43.9%) | 0.0136 |  |
| Hormonal contraceptives | 1024 (31.9%) | 101 (46.5%) | <0.001 | 72 (46.2%) | <0.001 | 80 (34.8%) | 0.40 |  |
| Hormone replacement therapy | 119 (3.7%) | 10 (4.6%) | 0.62 | 9 (5.8%) | 0.27 | 21 (9.1%) | <0.001 |  |
| Progestogens | 277 (8.6%) | 25 (11.5%) | 0.182 | 10 (6.4%) | 0.412 | 19 (8.3%) | 0.946 |  |
| Local estrogens | 65 (2.0%) | 11 (5.1%) | 0.0067 | <=5 | 0.357 | <=5 | 0.957 |  |
| GnRH agonists | 267 (8.3%) | 31 (14.3%) | 0.004 | 21 (13.5%) | 0.0358 | 27 (11.7%) | 0.0941 |  |
| **Prescription-level analgesics** |  |  |  |  |  |  |  |  |
| *NSAIDs* | 1750 (54.5%) | 157 (72.4%) | <0.001 | 104 (66.7%) | 0.00367 | 148 (64.3%) | 0.0046 |  |
| Number of prescriptions Median [Min, Max] | 2.00 [1.00, 73.0] | 3.00 [1.00, 28.0] | <0.001 | 3.00 [1.00, 34.0] | 0.001 | 3.00 [1.00, 47.0] | <0.001 |  |
| Number of days dispensed Median [Min, Max] | 24.0 [2.00, 2320] | 43.0 [3.00, 546] | <0.001 | 37.5 [3.00, 811] | 0.001 | 40.0 [4.00, 1430] | <0.001 |  |
| *Opioids* | 1961 (61.1%) | 177 (81.6%) | <0.001 | 107 (68.6%) | 0.0712 | 170 (73.9%) | <0.001 |  |
| Number of prescriptions Median [Min, Max] | 2.00 [1.00, 363] | 3.00 [1.00, 356] | <0.001 | 3.00 [1.00, 274] | <0.001 | 2.00 [1.00, 216] | <0.001 |  |
| Number of days dispensed Median [Min, Max] | 8.00 [1.00, 3610] | 17.0 [1.00, 2070] | <0.001 | 16.0 [1.00, 2510] | <0.001 | 12.0 [1.00, 2420] | <0.001 |  |
| **Psychotropics** |  |  |  |  |  |  |  |  |
| Anticonvulsants | 69 (2.1%) | 12 (5.5%) | 0.00324 | 11 (7.1%) | <0.001 | 16 (7.0%) | <0.001 |  |
| Antidepressants (other than SRIs, 2 years before) | 183 (5.7%) | 66 (30.4%) | <0.001 | 29 (18.6%) | <0.001 | 23 (10.0%) | 0.012 |  |
| Antidepressants (other than SRIs, 5 years before) | 282 (8.8%) | 81 (37.3%) | <0.001 | 44 (28.2%) | <0.001 | 39 (17.0%) | <0.001 |  |
| SRIs (2 years before) | 157 (4.9%) | 142 (65.4%) | <0.001 | 64 (41.0%) | <0.001 | 88 (38.3%) | <0.001 |  |
| SRIs (5 years before) | 297 (9.2%) | 156 (71.9%) | <0.001 | 77 (49.4%) | <0.001 | 101 (43.9%) | <0.001 |  |
| Benzodiazepines | 448 (13.9%) | 103 (47.5%) | <0.001 | 65 (41.7%) | <0.001 | 87 (37.8%) | <0.001 |  |
