# Supplementary material for: The Association Between Endometriosis Treatments and Depression and/or Anxiety in a Population-Based Pathologically Confirmed Cohort of People with Endometriosis
Source: Womens Health Rep (New Rochelle). 2023 Nov 20;4(1):551–61. doi: 10.1089/whr.2023.0068 (PMC10664573; doi:10.1089/whr.2023.0068)
Supplement: Supplemental data [file Suppl_TableS2-S4.zip › SupplementaryTable4.docx]

**Supplementary Table 4**. Endometriosis treatments received after the index surgery, stratified by the presence or absence of comorbid depression and/or anxiety in the two years prior to the index surgery. (GnRH – Gonadotropin Releasing Hormone, NSAID – Non-steroidal Anti-inflammatory Drug, SRI – Serotonin Reuptake Inhibitor)

|  | **No Mental Illness (reference group) (N=3212)** | **Depression only (N=217)** | **P-value** | **Anxiety only (N=156)** | **P-value** | **Code50B only (N=230)** | **P-value** |
| --- | --- | --- | --- | --- | --- | --- | --- |
| Reoperation for Endometriosis | 712 (22.2%) | 64 (29.5%) | 0.018 | 47 (30.1%) | 0.026 | 49 (21.3%) | 0.81 |
| **Physician visits – 3-24 months post-surgery** |  |  |  |  |  |  |  |
| *Pelvic Pain* | 648 (20.2%) | 79 (36.4%) | <0.001 | 45 (28.8%) | 0.012 | 61 (26.5%) | 0.027 |
| Number of visits for pain |  |  |  |  |  |  |  |
| Median [Min, Max] | 1.00 [1.00, 20.0] | 2.00 [1.00, 30.0] | 0.002 | 2.00 [1.00, 14.0] | 0.099 | 2.00 [1.00, 12.00] | 0.116 |
| *Endometriosis* | 625 (19.5%) | 53 (24.4%) | 0.091 | 39 (25.0%) | 0.11 | 37 (16.1%) | 0.243 |
| Number of visits for endometriosis |  |  |  |  |  |  |  |
| Median [Min, Max] | 2.00 [1.00, 20.0] | 2.00 [1.00, 25.0] | 0.383 | 2.00 [1.00, 16.0] | 0.040 | 2.00 [1.00, 9.00] | 0.402 |
| **Physician visits – 2-5 years post-surgery** |  |  |  |  |  |  |  |
| *Pelvic Pain* | 643 (20.0%) | 78 (35.9%) | <0.001 | 47 (30.1%) | 0.00314 | 51 (22.2%) | 0.483 |
| Number of visits for pain |  |  |  |  |  |  |  |
| Median [Min, Max] | 2.00 [1.00, 45.0] | 2.00 [1.00, 77.0] | 0.049 | 2.00 [1.00, 17.0] | 0.854 | 2.00 [1.00, 9.00] | 0.249 |
| *Endometriosis* | 554 (17.2%) | 46 (21.2%) | 0.165 | 37 (23.7%) | 0.0492 | 35 (15.2%) | 0.484 |
| Number of visits for endometriosis |  |  |  |  |  |  |  |
| Median [Min, Max] | 2.00 [1.00, 29.0] | 2.00 [1.00, 20.0] | 0.252 | 3.00 [1.00, 25.0] | 0.056 | 2.00 [1.00, 15.0] | 0.833 |
| **Hormonal medications (any prescription)** |  |  |  |  |  |  |  |
| Systemic estrogens | 322 (10.0%) | 28 (12.9%) | 0.215 | 12 (7.7%) | 0.415 | 29 (12.6%) | 0.255 |
| Estrogens and Progestogens (any combination) | 1211 (37.7%) | 89 (41.0%) | 0.368 | 71 (45.5%) | 0.060 | 88 (38.3%) | 0.922 |
| Hormonal contraceptives | 765 (23.8%) | 50 (23%) | 0.86 | 40 (25.6%) | 0.670 | 45 (19.6%) | 0.165 |
| Hormone replacement therapy | 446 (13.9%) | 39 (18.0%) | 0.12 | 31 (19.9%) | 0.048 | 43 (18.7%) | 0.055 |
| Progestogens | 314 (9.8%%) | 29 (13.4%) | 0.112 | 22 (14.4%) | 0.104 | 29 (12.6%) | 0.204 |
| Local estrogens | 451 (14.0%) | 44 (20.3%) | 0.0151 | 29 (18.6%) | 0.142 | 53 (23.0%) | <0.001 |
| GnRH agonists | 324 (10.1%) | 26 (12.0%) | 0.438 | 15 (9.6%) | 0.956 | 17 (7.4%) | 0.227 |
| **Prescription-level analgesics – 3-24 months post-surgery** |  |  |  |  |  |  |  |
| *NSAIDs* | 689 (21.5%) | 95 (43.8%) | <0.001 | 55 (35.3%) | <0.001 | 65 (28.3%) | 0.0198 |
| Number of prescriptions Median [Min, Max] | 1.00 [1.00, 16.0] | 2.00 [1.00, 16.0] | 0.007 | 1.00 [1.00, 9.00] | 0.759 | 1.00 [1.00, 16.0] | 0.433 |
| Number of prescription days dispensed  Median [Min, Max] | 15.0 [1.00, 547] | 21.0 [3.00, 450] | 0.016 | 20.0 [5.00, 342] | 0.353 | 30.0 [3.00, 435] | 0.066 |
| *Opioids* | 702 (21.9%) | 110 (50.7%) | <0.001 | 50 (32.1%) | 0.00388 | 73 (31.7%) | <0.001 |
| Number of prescriptions Median [Min, Max] | 1.00 [1.00, 228] | 3.00 [1.00, 190] | <0.001 | 2.00 [1.00, 82.0] | 0.005 | 2.00 [1.00, 63.0] | <0.001 |
| Number of prescription days dispensed  Median [Min, Max] | 8.00 [1.00, 590] | 23.0 [2.00, 557] | <0.001 | 16.5 [2.00, 575] | 0.031 | 17.0 [1.00, 551] | <0.001 |
| **Prescription-level analgesics – 2-5 years post-surgery** |  |  |  |  |  |  |  |
| *NSAIDs* | 1062 (33.1%) | 101 (46.5%) | <0.001 | 64 (41.0%) | 0.0486 | 94 (40.9%) | 0.0188 |
| Number of prescriptions Median [Min, Max] | 1.00 [1.00, 33.0] | 2.00 [1.00, 18.0] | <0.001 | 1.00 [1.00, 34.0] | 0.688 | 2.00 [1.00, 66.0] | 0.004 |
| Number of prescription days dispensed Median [Min, Max] | 20.0 [1.00, 1060] | 38.0 [5.00, 929] | <0.001 | 20.5 [2.00, 1020] | 0.995 | 25.0 [5.00, 1010] | 0.021 |
| *Opioids* | 1033 (32.2%) | 129 (59.4%) | <0.001 | 73 (46.8%) | <0.001 | 105 (45.7%) | <0.001 |
| Number of prescriptions Median [Min, Max] | 2.00 [1.00, 776] | 3.00 [1.00, 2750] | <0.001 | 2.00 [1.00, 132] | 0.002 | 2.00 [1.00, 169] | <0.001 |
| Number of prescription days dispensed  Median [Min, Max] | 8.00 [1.00, 1140] | 25.0 [1.00, 1110] | <0.001 | 22.0 [2.00, 1100] | 0.011 | 12.0 [1.00, 1110] | <0.001 |
| **Psychotropics (any prescription)** |  |  |  |  |  |  |  |
| Anticonvulsants | 478 (14.9%) | 78 (35.9%) | <0.001 | 41 (26.3%) | <0.001 | 55 (23.9%) | <0.001 |
| Antidepressants (other than SRIs) | 669 (20.8%) | 117 (53.9%) | <0.001 | 65 (41.7%) | <0.001 | 85 (37.0%) | <0.001 |
| SRIs | 801 (24.9%) | 178 (82.0%) | <0.001 | 83 (53.2%) | <0.001 | 117 (50.9%) | <0.001 |
| Benzodiazepines | 1007 (31.4%) | 135 (62.2%) | <0.001 | 84 (53.8%) | <0.001 | 135 (58.7%) | <0.001 |
