# Supplementary material for: The Association Between Endometriosis Treatments and Depression and/or Anxiety in a Population-Based Pathologically Confirmed Cohort of People with Endometriosis
Source: Womens Health Rep (New Rochelle). 2023 Nov 20;4(1):551–61. doi: 10.1089/whr.2023.0068 (PMC10664573; doi:10.1089/whr.2023.0068)
Supplement: Supplemental data [file Suppl_TableS5.docx]

**Supplementary Table 5.** Canadian Classification of Health Interventions (CCI) surgical procedure codes^27^

| **CCI code** | **Description** | **Assumption of common endometriosis procedure** |
| --- | --- | --- |
| 1.RM.89 | Hysterectomy | Hysterectomy (abdominal, vaginal, laparoscopic) |
| 2.RM.70.X | Inspection, uterus and surrounding structures | Diagnostic laparoscopy |
| 2.OT.70 | Inspection, uterus and surrounding structures | Diagnostic laparoscopy |
| 2.RM.71.X and 2.OT.71 | Biopsy of uterus and surrounding structures, biopsy of abdominal cavity | Biopsy of abdominal cavity |
| 1.OT.72 | Adhesiolysis, abdominal | Adhesiolysis/lysis of adhesions |
| 1.RD.72 | Adhesiolysis, tubal | Adhesiolysis/lysis of adhesions surrounding Fallopian tube |
| 1.RM.87.X | Excision, partial uteruuterine nerves and surrounding structures including excision of aberrant endometrial tissue | Excision of endometriosis |
| 1.RM.59.X | Ablation/cautery of endometriosis | Ablation/cautery of endometriosis |
| 1.RB.87.X | Excision partial, ovary | Ovarian cystectomy |
| 1.RB.72 | Manual rupture and drainage of ovarian cyst | Drainage of ovarian cyst |
| 1.RB.89.X | Excision total, ovary | Oophorectomy |
| 1.RF.89.X, 1.RD.89.X | Salpingoophorectomy | Salpingoophorectomy |
| 1.RF.87.X | Excision partial, fallopian tube | Salpingectomy |
| 1.RF.89.X | Excision total, fallopian tube | Salpingectomy |
| 1.RN.87.X | Trachelectomy | Total trachelectomy |
| 1.RN.89.X | Partial trachelectomy | Partial trachelectomy |
| 1.BF.59 | Uterine nerve ablation, uterosacral nerve ablation | Presacral neurectomy, uterosacral nerve ablation |
