# Supplementary material for: The Association Between Endometriosis Treatments and Depression and/or Anxiety in a Population-Based Pathologically Confirmed Cohort of People with Endometriosis
Source: Womens Health Rep (New Rochelle). 2023 Nov 20;4(1):551–61. doi: 10.1089/whr.2023.0068 (PMC10664573; doi:10.1089/whr.2023.0068)
Supplement: Supplemental data [file Suppl_TableS6.docx]

**Supplementary Table 6:** Medication covariates and relevant Anatomical Therapeutic Classification (ATC) codes**,** Drug Identification Numbers (DIN) and Product Identification Numbers (PIN).

|  | Covariate |  |  | Anatomical Therapeutic Classification (ATC) | DINPIN |
| --- | --- | --- | --- | --- | --- |
| **Hormonal Medications** |  |  |  |  |  |
|  | Systemic Estrogens Only |  |  | ATC 4 (G03CA, G03CB, L02AA) |  |
|  | Estrogens and Progestogens  (Any combination) | Hormonal Contraceptives | Estrogen and progestogens, combined prescription | ATC 4 (G02BB, G03AA, G03AB) |  |
|  |  | Hormone Replacement Therapy | Estrogen and progestogens, combined prescription | ATC 4 (G03FA, G03FB) |  |
|  |  |  | Combination of systemic estrogens and progestogens | ATC 4 (G03CA, G03CB, L02AA)  ATC 4 (G03AC, G03AD, G03DA, G03DB, G03DC)  ATC 4 (G02BA) |  |
|  | Progestogens Only |  | Progestogens only prescription or Mirena Intrauterine Device (IUD) | ATC 4 (G03AC, G03AD, G03DA, G03DB, G03DC)  ATC 4 (G02BA) |  |
|  | Local Estrogens |  |  | ATC 4 (G03CA, G03CB) | 2089, 727369, 2043440, 2168898, 2241332, 2325462, 441295 |
|  | Gonadotropin Releasing Hormone (GnRH) Agonists |  |  | ATC 4 (G03XA, G02GB)  ATC 5 (H01CA02) |  |
| **Prescription-level Analgesics** |  |  |  |  |  |
|  | Non-steroidal anti-inflammatory drugs (NSAIDs) |  |  | ATC 4 (C01EB, M01AB, M01AE, M01AH, N02BA) |  |
|  | Opioids |  |  | ATC 4 (N01AH, N02AA, N02AB, N02AX, N02BA, N02BE, N07BC, R05DA) |  |
| **Psychotropics** |  |  |  |  |  |
|  | Anticonvulsants |  |  | ATC 5 (N03AX12, N03AXX14, N03AX16) |  |
|  | Antidepressants |  |  | ATC 4 (N06AA, N06AB, N06AF, N06AG, N06AX) |  |
|  | Serotonin Reuptake Inhibitors |  |  | ATC 4 (G02BB, G03AA, G03AB, G03FA, G03FB) |  |
|  | Benzodiazepines |  |  | ATC 4 (N03AE, N05AA, N05AH, N05BA, N05CD, N05CF) |  |
