# Supplementary material for: The Association Between Endometriosis Treatments and Depression and/or Anxiety in a Population-Based Pathologically Confirmed Cohort of People with Endometriosis
Source: Womens Health Rep (New Rochelle). 2023 Nov 20;4(1):551–61. doi: 10.1089/whr.2023.0068 (PMC10664573; doi:10.1089/whr.2023.0068)
Supplement: Supplemental data [file Suppl_FigureS1.docx]

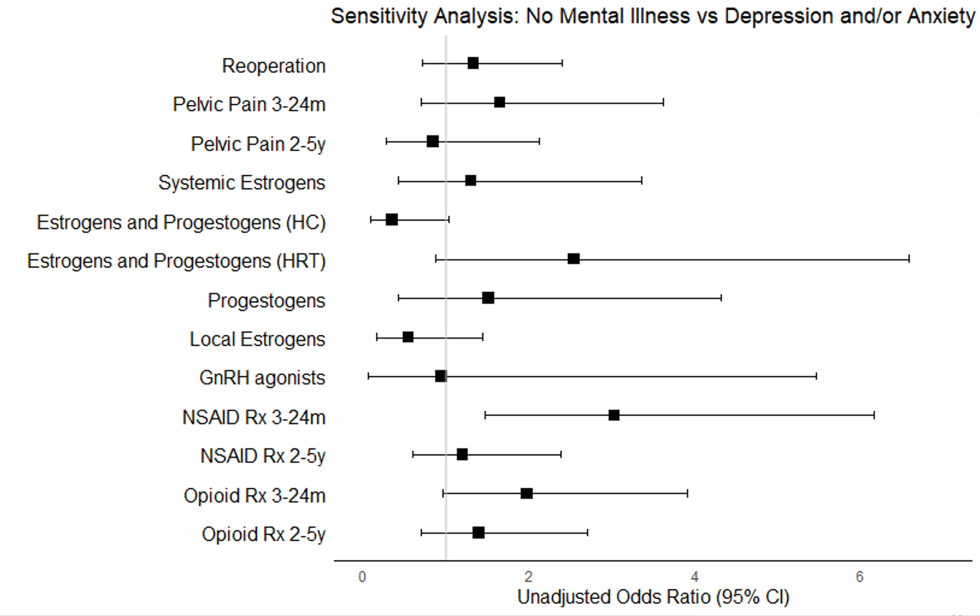

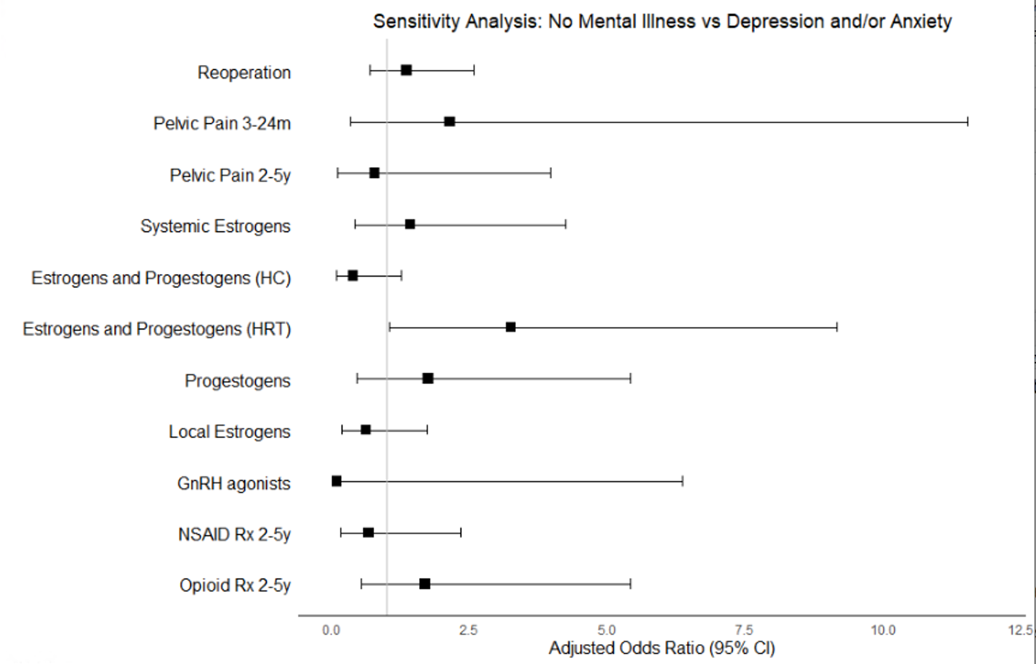


Supplementary Figure 1. Sensitivity analysis including only those with no history of endometriosis based on physician visits, previous surgery, and indication for the index surgery. Individuals with no mental illness were compared to those with depression and/or anxiety for various endometriosis treatments. The odds ratios were adjusted for age, income quintile, surgical indication (including pain, mass/suspected cancer, cysts, and infertility), fibroids, surgical approach, hysterectomy, bilateral salpingoophorectomy, and other procedures. NSAID Rx and Opioid Rx in the 3-24m time period were removed from the adjusted figure for readability, as they had large confidence intervals. Abbreviations: Rx – Prescription; HC – hormonal contraception; HRT – hormone replacement therapy; GnRH – gonadotropin-releasing hormone; NSAID – non-steroidal anti-inflammatory drug.
