# Supplementary material for: The Association Between Endometriosis Treatments and Depression and/or Anxiety in a Population-Based Pathologically Confirmed Cohort of People with Endometriosis
Source: Womens Health Rep (New Rochelle). 2023 Nov 20;4(1):551–61. doi: 10.1089/whr.2023.0068 (PMC10664573; doi:10.1089/whr.2023.0068)
Supplement: Supplemental data [file Suppl_TableS7.docx]

Supplementary Table 7. Odds ratios for the sensitivity analysis comparing treatment outcomes for those with no mental illness and those with depression and/or anxiety. Sensitivity cohort included only those who had no previous physician visits for endometriosis, no clinical indication for endometriotic cysts or other endometriosis, indications for surgery that were not endometriosis, and no endometriosis surgery in the 45 days before index surgery. n=417; 358 with no mental illness and 59 with depression and/or anxiety. Odds ratios were adjusted for: age, income quintile, clinical indication of pain, mass/suspected cancer, cyst, or infertility, fibroids, surgical approach, hysterectomy, BSO, or other procedure.

|  | OR | 95%CI | aOR | 95%CI | # of total events | # of events in those with dep/anx |
| --- | --- | --- | --- | --- | --- | --- |
| Reoperation | 1.33 | 0.71-2.40 | 1.36 | 0.70-2.57 | 107 | 18 |
| Pelvic Pain  (3-24m) | 1.65 | 0.70-3.62 | 2.14 | 0.35-11.54 | 48 | 9 |
| Pelvic Pain  2-5y) | 0.84 | 0.28-2.12 | 0.78 | 0.10-3.96 | 43 | 5 |
| Systemic Estrogen | 1.30 | 0.42-3.35 | 1.43 | 0.42-4.25 | 31 | 5 |
| Estrogen and Progestogens combined (HC) | 0.35 | 0.08-1.03 | 0.39 | 0.09-1.27 | 52 | 3 |
| Estrogens and Progestogens combined (HRT) | 2.54 | 0.87-6.58 | 3.25 | 1.05-9.17 | 23 | 6 |
| Progestogens | 1.51 | 0.42-4.31 | 1.75 | 0.46-5.41 | 22 | 4 |
| Local Estrogens | 0.54 | 0.16-1.43 | 0.62 | 0.18-1.74 | 49 | 4 |
| GnRH agonists | 0.94 | 0.05-5.46 | 0.10 | 0.05-6.37 | 8 | 1 |
| NSAIDs  (3-24m) | 3.03 | 1.46-6.15 | 21.7 | 5.09-122.18 | 58 | 15 |
| NSAIDs  (2-5y) | 1.20 | 0.59-2.38 | 0.67 | 0.17-2.35 | 95 | 14 |
| Opioids  (3-24m) | 1.97 | 0.96-3.91 | 4.76 | 1.31-19.0 | 76 | 15 |
| Opioids  (2-5y) | 1.39 | 0.70-2.70 | 1.69 | 0.53-5.41 | 108 | 17 |
